# Interdigital Pedal Erythrasma treated with one‐time dose of oral clarithromycin 1 g: Two case reports

**DOI:** 10.1002/ccr3.2712

**Published:** 2020-02-06

**Authors:** Mohammad Junayed Khan

**Affiliations:** ^1^ Montefiore Mount Vernon Hospital Mount Vernon New York

**Keywords:** clarithromycin, erythrasma, interdigital maceration, superficial foot infection

## Abstract

The following are the first detailed cases of Interdigital Pedal Erythrasma successfully treated with a one‐time dose of oral clarithromycin 1 g. This is ideal for patients who failed topical therapy or have mechanical or psychosocial restrictions. Treatment provides better compliance, less gastric side effects, and lower treatment costs than oral alternatives.

## Graphical abstract

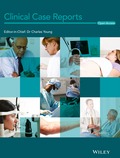

## 1 INTRODUCTION

Interdigital Pedal Erythrasma (IPE) is a mild, localized superficial infection between the toes caused by *Corynebacterium minutissimum*. *Corynebacterium minutissimum* is a member of the normal skin flora, which usually grows in moist and occluded areas such as the axillae, inframammary areas, intergluteal and crural folds, and interdigital regions of the foot.^1^ However, Erythrasma most commonly presents in the interspaces of the foot.^1^ Interdigital Pedal Erythrasma is often characterized by erythematous plaques, scaly patches, interdigital maceration, and in more severe cases, vesicles, blistering, and fissuring.^1^, ^2^ Interdigital Pedal Erythrasma is often misdiagnosed as interdigital tinea pedis, but an interdigital *C minutissimum* infection often coexists with other bacteria, dermatophytes and yeasts.^3^ Diagnosis of IPE is typically made by a proper Wood's lamp examination, which reveals coral‐red fluorescence in the affected interspaces, and microscopic examination with potassium hydroxide and gram staining.^2^ Cultures are usually difficult and unsuccessful and are not considered necessary for diagnosis of IPE.^4^ Currently, dermatologists and foot specialists prescribe topical antibiotics such as erythromycin, clindamycin, or mupirocin to treat the causative organism, *C minutissimum*, involved in IPE.

When topical antibiotic therapy fails in treating Interdigital Pedal Erythrasma, whether for medical or psychosocial reasons, oral antibiotics such as erythromycin, tetracycline, and clarithromycin must be considered. Patients will be more compliant with a one‐time dose of 1‐g oral clarithromycin as opposed to the one 250 mg tablet, taken four times daily, for 14 days dosing schedules of both erythromycin and tetracycline. Clarithromycin has a safer adverse reaction profile than tetracycline and can be safely used on pediatric patients as it does not cause tooth discoloration in children.^5^ While clarithromycin and erythromycin cause similar adverse reactions, clarithromycin causes less gastric side effects while having higher bioavailability, longer half‐life, and broader spectrum of antimicrobial activity compared to erythromycin.^6^ Comparing the oral options in cost, the total cost of treatment of this clarithromycin regimen is only $1.20 USD, significantly less than erythromycin costing $22.40 USD and tetracycline costing $11.20 USD.^5^ Therefore, compared to other oral antibiotic regimens in the treatment of IPE, a one‐time oral dose of clarithromycin 1 g allows for better compliance, less gastric side effects, and substantially lower treatment costs.

There is scarce information published on the use of a one‐time dose of clarithromycin 1 g to treat Erythrasma. There are two published studies, with only four reported patients treated in total, in the literature detailing the success of a one‐time dose of clarithromycin 1 g to treat inguinal Erythrasma, none of which involved IPE.^5^, ^6^ The following case reports detail the first documented cases of IPE treated with a one‐time dose of oral clarithromycin 1 g. In this study, two patients with confirmed Interdigital Pedal Erythrasma without concomitant tinea pedis were no longer able to apply topical antibiotics to the affected interspaces, either due to failure of topical therapy or the physical inability to apply topical antibiotics to the affected interspaces.

## 2 CASE 1

A 60‐year‐old African American female with no past medical history presents with interdigital maceration in her right 4th interspace. The maceration has been present for 7 months, and she had been previously diagnosed with Erythrasma of her right 4th interspace. She states she was educated on proper foot hygiene to keep her interspaces dry and has been applying betadine in her affected interspace twice a day since first being diagnosed with Erythrasma. She also applied topical erythromycin 2%, then topical clindamycin 1% gel, both of which failed. Wood's lamp examination showed coral‐red fluorescence, and samples from the macerated interspace were sent for 20% potassium hydroxide (KOH) preparation and tissue culture. The KOH examination was negative for fungal organisms, and the tissue culture was positive for *Corynebacterium* species.

The patient was treated with 1 g of oral clarithromycin administered as a single dose of two 500‐mg tablets. She was also re‐educated on proper foot hygiene to keep her interspaces dry and advised to continue applying betadine in her interspaces as she has been doing for the past 7 months. The patient did not experience any adverse reactions associated with the medication. After 2 weeks, the patient's interdigital maceration in her right 4th interspace had decreased, but Wood's lamp examination still displayed coral‐red fluorescence and a repeat tissue culture examination remained positive for *Corynebacterium* species. At her 4‐week follow‐up, there were no clinical signs of interdigital maceration or Erythrasma noted. Wood's lamp examination did not fluorescence, and a repeat tissue culture examination was negative. At her 6‐week follow‐up, there remained no clinical or microbiological signs of Erythrasma evidenced by a negative Wood's lamp examination and negative tissue culture.

## 3 CASE 2

An 80‐year‐old African American male with no past medical history presents with interdigital maceration in his left 4th interspace. The maceration has been present for 2 months, and he was previously diagnosed with Erythrasma and prescribed topical erythromycin 2%. However, he admits to not applying it regularly due to his low back pain and inability to bend over. He states he was educated on proper foot hygiene to keep his interspaces dry. Wood's lamp examination showed coral‐red fluorescence, and samples from the macerated interspace were sent for 20% potassium hydroxide (KOH) preparation and tissue culture. The KOH examination was negative for fungal organisms, and the tissue culture was positive for *Corynebacterium* species.

The patient was treated with 1 g of oral clarithromycin administered as a single dose of two 500‐mg tablets. He was also re‐educated on proper foot hygiene to keep his interspaces dry. The patient did not experience any adverse reactions associated with the medication. After 2 weeks, the patient had no clinical signs of interdigital maceration in the left 4th interspace, and a Wood's lamp examination was negative for coral‐red fluorescence. A repeat tissue culture examination was negative for *Corynebacterium* species. At his 4‐week follow‐up, there were no clinical signs of interdigital maceration or Erythrasma noted. Wood's lamp examination did not fluorescence, and a repeat tissue culture examination remained negative.

## 4 CONCLUSION

Previously, there were only a few documented cases of Erythrasma being treated with a one‐time dose of 1‐g oral clarithromycin, which were all cases of inguinal Erythrasma.^5^, ^6^ These case reports depict two instances in which Interdigital Pedal Erythrasma, without concomitant interdigital tinea pedis, was successfully treated with a one‐time dose of 1‐g oral clarithromycin: these are the first documented cases of IPE successfully treated in such a way. For both patients, the antibiotic regimen did not cause any adverse health effects, was readily available for a low cost, and was an easy treatment regimen to comply with. A one‐time dose of 1‐g oral clarithromycin may be particularly useful in patients who are unable to apply a topical antibiotic or antiseptic due to mental disabilities or common mechanical restrictions caused by poor eyesight, obesity, or chronic back pain. In addition, this treatment regimen may be useful in patients who have failed topical antibiotic therapy for treating IPE or for noncomplaint patients in general. The two documented cases depict a clinical and microbiological cure between 2 and 4 weeks after receiving the one‐time dose of 1 g clarithromycin, which agrees with the time to cure of a case report of this antibiotic regimen in treating Erythrasma in the thigh.^5^ However, further studies are necessary to investigate the clinical efficacy of one‐time dose of 1 g clarithromycin as these reports consist of a small sample size with a relatively short follow‐up period of 2 weeks after clinical and microbiological cure. Future studies should investigate patients with Interdigital Pedal Erythrasma with a potential concomitant dermatophyte infection, or with comorbidities such as diabetes mellitus or peripheral arterial disease: These factors may possibly diminish the efficacy of a one‐time dose of 1 g oral clarithromycin, which remains to be determined.

## CONFLICT OF INTEREST

None declared.

## AUTHOR CONTRIBUTION

MJK: is the primary and sole author who contributed to the content of this publication in its entirety.
